# Analysis of sputum microbial metagenome in COPD based on exacerbation frequency and lung function: a case control study

**DOI:** 10.1186/s12931-022-02246-9

**Published:** 2022-11-19

**Authors:** Wei Li, Bingbing Wang, Min Tan, Xiaolian Song, Shuanshuan Xie, Changhui Wang

**Affiliations:** 1 Department of Geriatrics, Shanghai Tenth People’s Hospital, School of Medicine, Tongji University, Shanghai, 200072 China; 2 Department of Respiratory Medicine, Shanghai Tenth People’s Hospital, School of Medicine, Tongji University, Shanghai, 200072 China

**Keywords:** Chronic obstructive pulmonary disease (COPD), Metagenomic next-generation sequencing, Sputum, Microbiome, Exacerbation frequency, Lung function

## Abstract

**Background:**

The role of the sputum microbiome in chronic obstructive pulmonary disease (COPD) progression remains elusive. As the advent of the new culture-independent microbial sequencing technique makes it possible to disclose the complex microbiome community of the respiratory tract. The aim of this study was to use metagenomic next-generation sequencing (mNGS) to confirm whether there are differences in sputum microbiome of COPD between different exacerbation frequencies and lung function.

**Methods:**

Thirty-nine COPD patients were divided into a frequent exacerbators (FE) group (n = 20) and a non-frequent exacerbators (NFE) (n = 19) group according to their exacerbation history, or a mild group (FEV_1_/pre ≥ 50%, n = 20) and a severe group (FEV_1_/pre < 50%, n = 19) according to the lung function. Sputum was collected during their stable phase, followed by DNA extraction, untargeted metagenomic next-generation sequencing (mNGS) and bioinformatic analysis.

**Results:**

mNGS identified 3355 bacteria, 71 viruses and 22 fungi at the specie level. It was found that Shannon index and Simpson index in FE group was lower than that in NFE group (p = 0.005, 0.008, respectively) but similar between mild and severe groups. Out of top 10 bacteria taxa, Veillonella, Fusobacterium and Prevotella jejuni had a higher abundance in NFE group, Rothia had a higher abundance in mild group. Linear discriminant analysis revealed that many bacterial taxa were more abundant in NFE group, and they mostly belonged to Actinobacteria, Bacteroidetes and Fusobacteria phyla. Frequency of exacerbations was also found to be negatively correlated with alpha diversity (with Shannon index, r = − 0.423, p = 0.009; with Simpson index, r = − 0.482, p = 0.002). No significant correlation was observed between alpha diversity and FEV_1_/pre.

**Conclusions:**

Microbiome diversity in FE group was lower than that in NFE group. There was a significant difference in microbiome taxa abundance between FE and NFE groups, or mild and severe groups. These findings demonstrated that sputum microbiome community dysbiosis was associated with different exacerbation frequencies and lung function in stable COPD.

**Supplementary Information:**

The online version contains supplementary material available at 10.1186/s12931-022-02246-9.

## Background

Chronic obstructive pulmonary disease (COPD) is a common chronic disease characterized by persistent respiratory symptoms and airflow limitation. COPD is the third leading cause of death in the world, responsible for approximately 6% of total deaths [1]. The impact of COPD on mortality and morbidity will persist due to the aging population and urbanization. Mounting evidence has shown that dysbiosis of pulmonary microbiota participates in COPD progress [2]. Exacerbation frequency and lung function are two major indexes of COPD. The former is related to airflow limitation, symptoms and health-related quality of life impairment [3, 4], and the latter is an index revealing severity of airflow limitation. Till now, the difference in sputum microbiome of COPD between different exacerbation frequencies or lung function is still poorly understood.

The primary aim of the present study was using Metagenomic Next-Generation Sequencing (mNGS), also known as shotgun metagenomic sequencing, to confirm whether there are differences in sputum microbiome of COPD between different exacerbation frequencies and lung function.

## Methods

### Patient selection and grouping

Patients who used to seek hospital treatment for COPD exacerbation in the Department of Respiratory Medicine of Shanghai Tenth People’s Hospital (Shanghai, China) were interviewed by telephone, and those who met with the inclusion criteria and agreed to participate in the research were invited to the said hospital for further medical checkups from April to May 2021. The inclusion criteria for stable COPD patients were: (a) symptoms of dyspnea, chronic cough or expectoration, and/or a history of exposure to risk factors for the disease; (b) the presence of a post-bronchodilator FEV_1_/FVC < 0.7; and (c) no symptomatic deterioration in recent 30 days [5]. Patients with malignancies, heart failure, cognitive disorders, and severe kidney or liver dysfunction were excluded. Basic demographic information, smoking history, exacerbation frequency in the last 1 year, modified Medical Research Council (mMRC) dyspnea scale and the COPD Assessment Test (CAT) were obtained. In addition, the participating patients were required to receive spirometry. Written informed consent was obtained from all participating patients before initiation of the investigation. The study protocol was approved by the Institutional Review Boards of the said hospital.

Patients with at least 2 treated exacerbation events in last 1 year were divided into a FE group, and patients with 1 or less treated exacerbation events in last 1 year were divided into a NFE group. In addition, the patients were also reclassified into a mild group (FEV_1_/pre ≥ 50%) and a severe group (FEV_1_/pre < 50%) according to the lung function.

### Sampling

Samples were the first expectorated sputum obtained in the morning. The patients were required to clean their mouth before expectorating the sputum spontaneously into a DNA-free tube.

### Nucleic acid extraction, library preparation and sequencing

DNA was extracted from all samples using a QIAamp® UCP Pathogen DNA Kit (Qiagen) following the manufacturer’s instructions. Human DNA was removed using Benzonase (Qiagen) and Tween20 (Sigma) [6]. 10 nanograms DNA samples were used for library construction through Nextera XT DNA Library Prep Kit (Illumina, San Diego, CA) [7]. Library was qualitatively assessed by Qubit dsDNA HS Assay kit, followed by High Sensitivity DNA kit (Agilent) on an Agilent 2100 Bioanalyzer. Library pools were then loaded onto an Illumina Nextseq 550Dx sequencer for 75 cycles of single-end sequencing to generate approximately 20 million reads for each library. For negative controls, we also prepared peripheral blood mononuclear cell(PBMC) samples with 10^5^ cells/mL from healthy donors in parallel with each batch, using the same protocol, and sterile deionized water was extracted alongside the specimens to serve as non-template controls (NTC) [7, 8]. DNA-free water went through DNA extraction and mNGS analysis as a blank control group to assess the degree of background contamination associated with DNA extraction kit and sequencing reagents together.

### Bioinformatics analysis

Trimmomatic [9] was used to remove low quality reads, adapter contamination, and duplicate reads, as well as those shorter than 50 bp. Low complexity reads were removed by Kcomplexity with default parameters [10]. Human sequence data were identified and excluded by mapping to a human reference genome (hg38) using Burrows-Wheeler Aligner software [11].

After alignment, multiple indicators were comprehensively evaluated to have the list of suspected microorganisms. Then, microbiota composition profiles were inferred from quality filtered forward reads using Kraken V.2.1.2 and Bracken V.2.6.2 with the k2_pluspf_20210517 database. The site by species counts and relative abundance tables were input into R-base V.4.1.0 for statistical analysis. Alpha diversity of the microbiota profile for each subject was assessed by group at the different level data using the Vegan package in R (version 2.5.7). Principal component analysis (PCA) ordinations were used to visualize the clustering of the samples based on the compositional profiles at genus or specie level. Different groups were assessed using permutational multi-variate analysis of variance (PERMANOVA) and PCA stat with the Vegan package in R (version 2.5.7). The result of PCOA was stated by the function of dudi.pco in ade4 package in R (version 1.7.18). Associations of specific microbial species or genus with patient parameters were identified using the linear discriminant analysis effect size (LEfSe) [12].

### Statistical analysis

SPSS Statistics 26 was used to perform statistical analysis of clinical variables comparison between groups. Results for categorical variables were expressed as absolute and relative frequencies while results for continuous variables were expressed as means and standard deviations (SD) if normally distributed or as medians and interquartile ranges (IQR) when the distribution was not normal. Chi squared test was conducted for all categorical variables. As for continuous variables, two independent-sample t-test was conducted for normally distributed continuous variables and Mann–Whitney U test was conducted for not normally distributed. Correlation analysis were performed through Spearman’s correlation analysis. p value < 0.05 was considered statistically significant.

## Results

### Patient characteristics

Altogether 39 stable COPD patients were enrolled in our study (Table 1). The range of the post-bronchodilator FEV_1_/FVC was 25.67–68.76%. According to their exacerbation history in the last 1 year, 19 fell in NFE group and 20 in FE group; according to their post-bronchodilator FEV_1_/pre value, 19 fell in severe COPD group and 20 in mild COPD group. Gender, age, smoking history and FEV_1_/FVC were comparable between FE and NFE groups, while BMI and FEV_1_/pre in NFE group were significantly higher than those in FE group (p = 0.038, 0.014, respectively). Gender, age and smoking history were also comparable between mild and severe groups, except that BMI in mild group was significantly higher than that in severe group (p = 0.041).

**Table 1:** Clinical characteristics of the study population

	All	NFE	FE	p value	Severe	Mild	p value
N	39	19	20		19	20	
Gender, Male (%)	29 (70.7%)	13 (68.4%)	16 (80%)	0.408	15 (78.9%)	14 (70%)	0.716
Age (years)							
Range	56–88	60–84	56–88		56–88	60–84	
Mean ± sd	69.18 ± 7.25	68.68 ± 6.84	69.65 ± 7.77	0.683	66 (61, 72)	70 (67, 74.75)	0.118
BMI							
Range	15.43–32.28	16.60–32.28	15.43–29.38		15.43–30.19	16.60–32.28	
Mean ± sd	23.74 ± 4.13	25.14 ± 4.03	22.41 ± 3.86	0.038	22.36 ± 4.23	25.05 ± 3.66	0.041
Smoking history							
Ever-smoker	27(69.2%)	12(63.2%)	15(75%)		14(73.7%)	13(65%)	
Never-smoker	12(30.8%)	7(36.8%)	5(25%)	0.423	5(26.3%)	7(35%)	0.557
FEV_1_/pre (%)							
Range	20.3–97.7	21.8–97.7	20.3–93.7		20.3–47.6	50.4–97.7	
Mean ± sd	49.62 ± 20.91	58.6(39.2, 69.4)	37.55(25.4, 54.7)	0.009	32.1(23.1, 39.2)	64(56.13, 70.88)	< 0.001
FEV_1_/FVC (%)							
Range	25.67–68.76	25.67–68.76	27.03–68.24		25.67–61.48	46.71–68.76	
Mean ± sd	48.36 ± 12.25	51.28 ± 11.94	45.59 ± 12.17	0.149	39.60 ± 10.08	56.69 ± 7.35	< 0.001

*FE* frequent exacerbators, *NFE* non-frequent exacerbators, *sd* standard deviation, *N* number, *BMI* body mass index, *FEV*_*1*_ forced expiratory volume in 1 s, *FEV*_*1*_*/pre* FEV_1_ percent predicted, *FVC* forced vital capacity. Non-normal distributed data were expressed as medians and interquartile ranges (IQR)

### Bacterial, viral and fungal composition

Deep metagenome sequencing totally identified 1012 bacteria, 27 viruses and 18 fungi at genus level, and 3355 bacteria, 71 viruses and 22 fungi at specie level.

The microbiome composition comparison of the blank control group and sample group was displayed in Additional file 1: Fig. S1. As shown in the Additional file 1: Fig. S1, the major microbiome components of blank control group were completely different from the sample group, which means that the degree of background contamination was low.

**Fig. 1:**
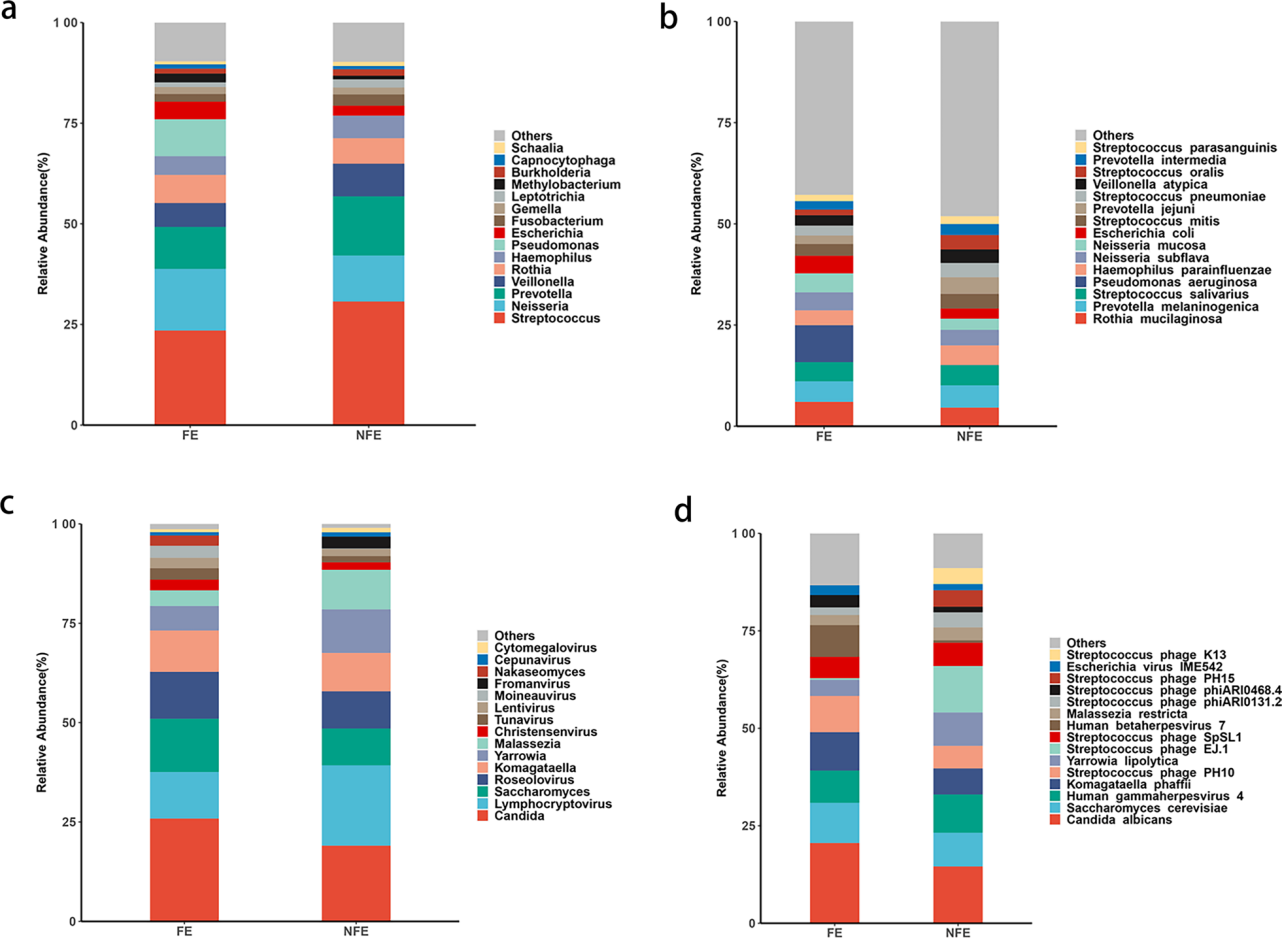
Relative abundance of top 15 microbiome taxa in stable COPD patients. **a** Relative abundance of top 15 bacterial genera between FE and NFE groups. **b** Relative abundance of top 15 bacterial species between FE and NFE groups. **c** Relative abundance of top 15 fungal and viral genera between FE and NFE group. **d** Relative abundance of top 15 fungal and viral species between FE and NFE groups

The compositions of the top 15 bacteria, viruses and fungi in the sputum of FE and NFE groups at both genus and specie level are shown in Fig. 1. The top 15 bacterial genera were Streptococcus, Neisseria, Prevotella, etc. Candida was the most dominant fungal genus in stable COPD. Lymphocryptovirus was the most dominant virus in stable COPD. In terms of species, the top 15 dominant bacteria were Rothia mucilaginosa, Prevotella melaninogenica, Streptococcus salivarius, etc. Candida albicans was the most abundant fungus while human gammaherpesvirus 4, also named Epstein-Barr virus, was the most abundant virus in stable COPD.

### Diversity comparison between groups

**Fig. 2:**
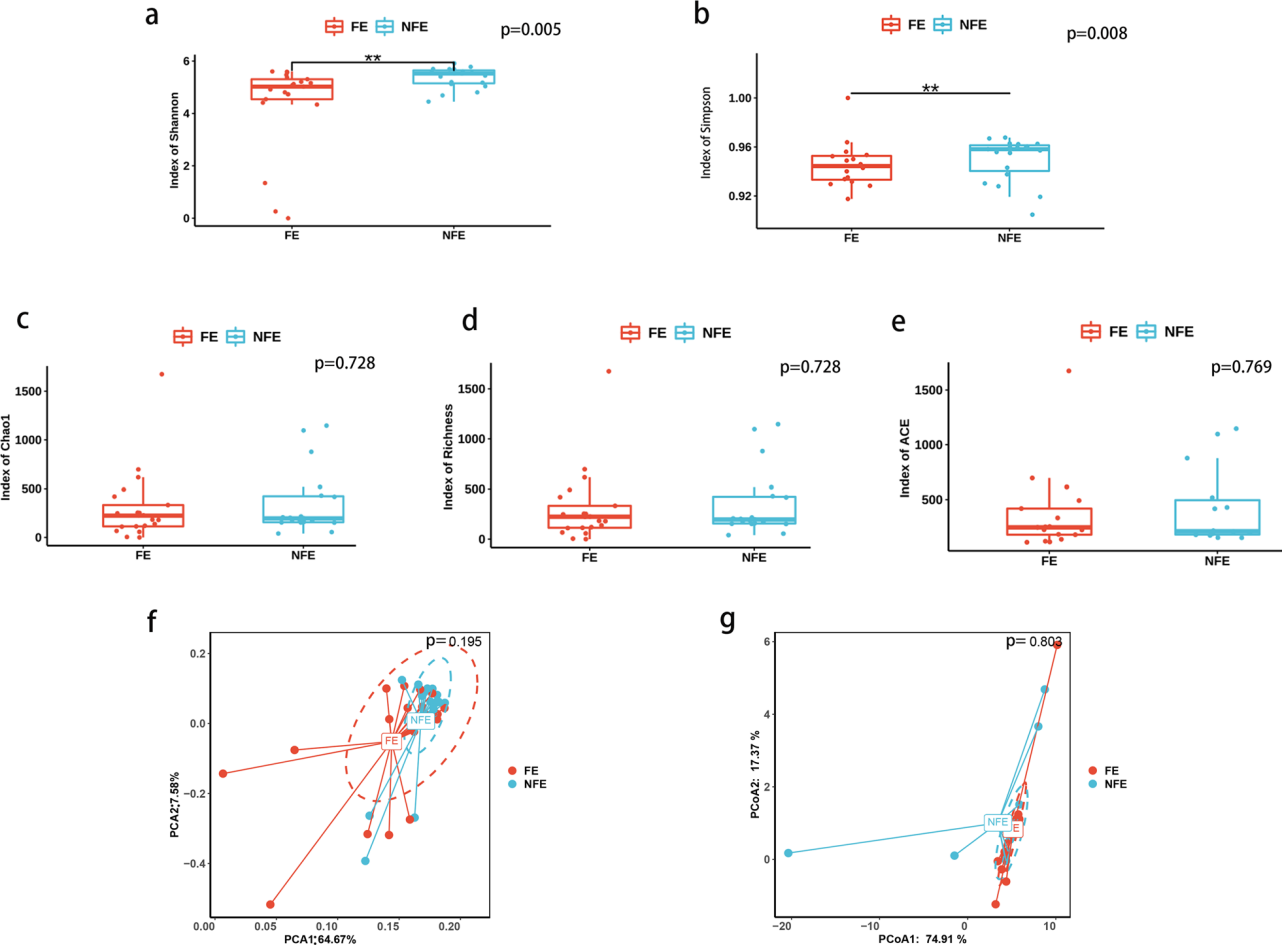
Alpha and beta diversity comparison between FE and NFE groups in stable COPD. **a** Shannon index of NFE were significantly higher than that of FE at specie level (p = 0.005); **b** Simpson index of NFE were significantly higher than that of FE at specie level (p = 0.008); Chao 1 index **(c)**, Richness index **(d)** and ACE index **(e)** were similar between FE and NFE groups. PCA **(f)** and PCoA **(g)** plot were presented in FE and NFE groups

We then compared alpha and beta diversity of the sputum microbiome between groups. Both Simpson index (p = 0.008) and Shannon index (p = 0.005) of NFE were significantly higher than those of FE at specie level (Fig. 2a, b). Chao1 index, Richness index and ACE index were similar between FE an NFE groups at specie level (Fig. 2c–e). Besides, PCA plot and PCoA showed no significant difference between NFE and FE groups (Fig. 2f, g). At genus level, no significant difference of diversity was detected between FE and NFE group. No significant difference in alpha and beta diversity was detected between mild and severe groups at genus or specie level.

### Differential taxa between groups

**Fig. 3:**
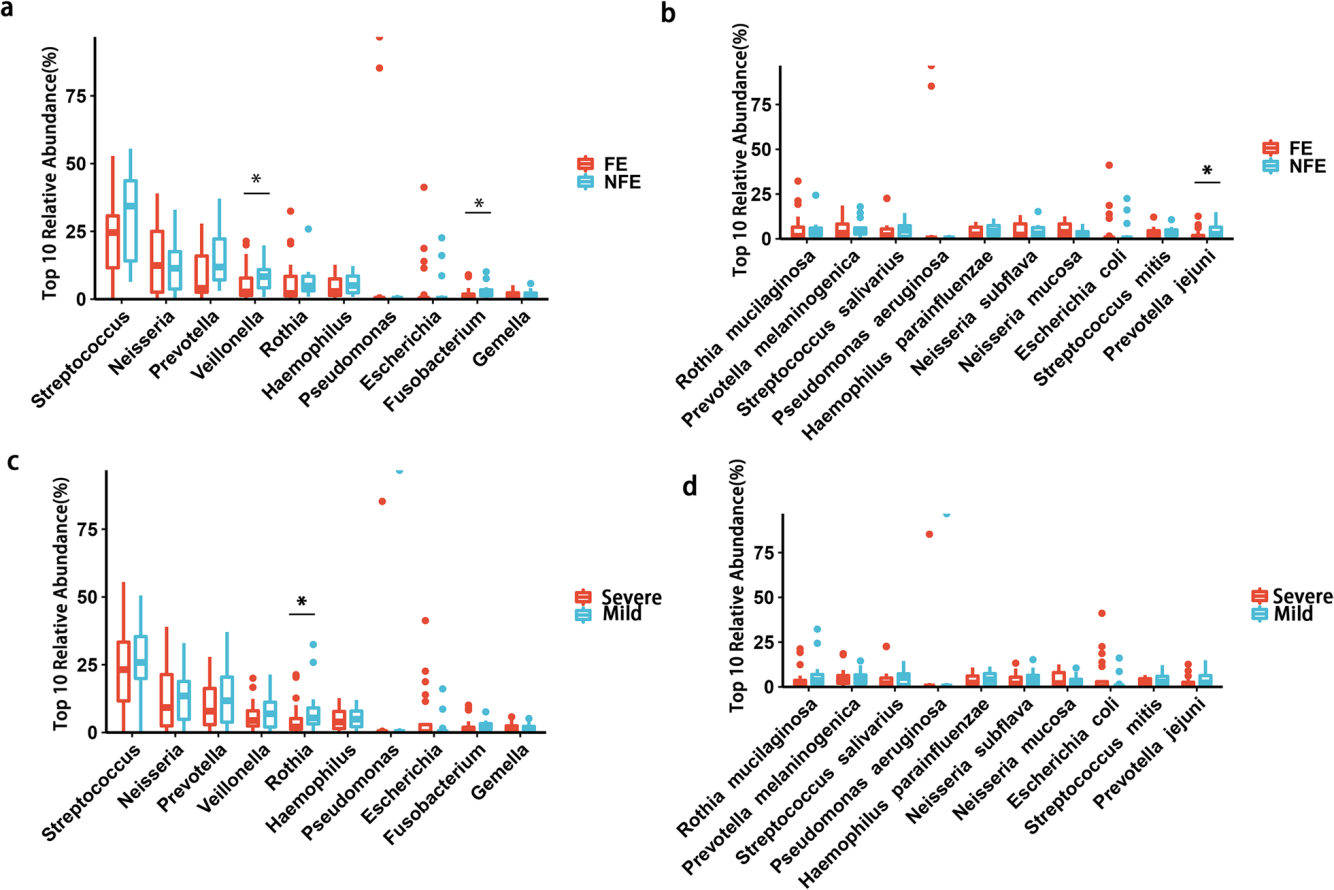
Abundance difference of top 10 bacterial taxa in stable COPD between groups. Mann–Whitney U test identified that **a** Veillonella, Fusobacterium had a higher relative abundance in NFE group compared with FE group. **b** Prevotella jejuni had a higher relative abundance in NFE group compared with FE group. **c** Rothia had a higher relative abundance in mild group compared with severe group **d **no significant differences of relative abundance of top 10 bacterial species were detected between mild and severe group

The relative abundance top 10 bacterial taxa was compared between FE and NFE, mild and severe group (Fig. 3). Mann–Whitney U test identified Veillonella, Fusobacterium and Prevotella jejuni were significantly more abundant in NFE group compared with FE group. Rothia were more abundant in mild group compared with severe group.

**Fig. 4:**
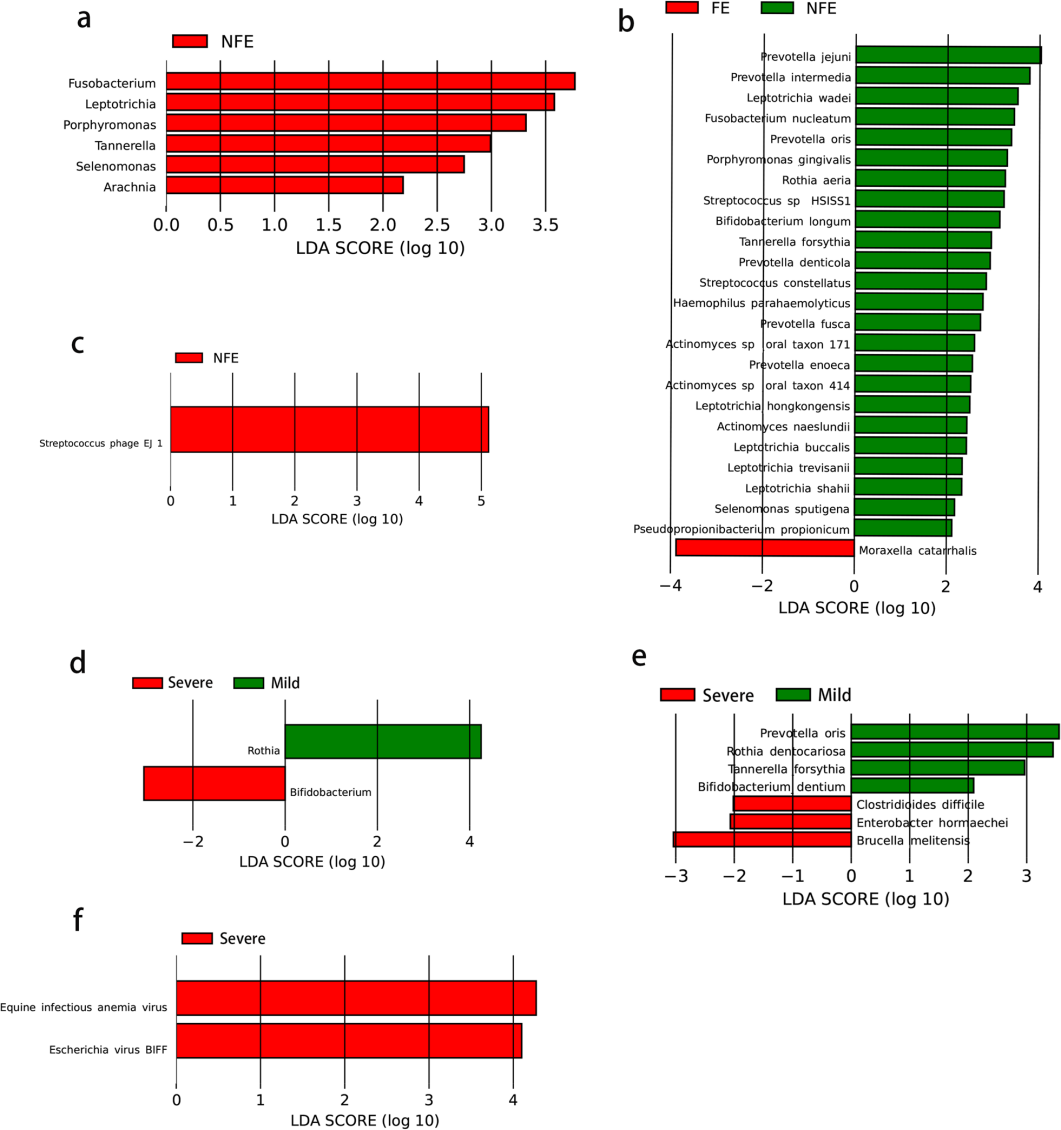
Linear discriminant analysis revealed differentially abundant bacterial taxa in stable COPD between groups. **a** Differences in bacterial genera between FE and NFE groups. **b** Differences in bacterial species between FE and NFE groups. **c** Difference in fungal and viral species between FE and NFE groups. **d** Differences in bacterial genera between mild and severe groups. **e** Differences in bacterial species between mild and severe groups. **f** Differences in fungal and viral species between mild and severe groups

A LEfSe analysis was also performed to identify differences in microbiome distribution between COPD FE and NFE subgroups. The significantly enriched genera and species (LDA > 2) in each group are displayed in Fig. 4. The relative abundance of Fusobacterium, Leptotrichia, Porphyromonas, etc. and Prevotella jejuni, Prevotella intermedia, Leptotrichia wadei, etc. was significantly increased in NFE group (Fig. 4a, b). While Moraxella catarrhalis was significantly increased in FE group (Fig. 4b). As for fungi and virus, Streptococcus phage EJ 1 was increased in NFE group (Fig. 4c). Differences in microbiome distribution between mild and severe groups were also identified. The relative abundance of Rothia, Prevotella oris, Rothia dentocariosa, Tannerella forsythia, etc. was significantly increased and the abundance of Bifidobacterium, Clostridioides difficile, Enterobacter hormaechei and Brucella melitensis was significantly decreased in mild COPD group (Fig. 4d, e). In terms of virus and fungi, the relative abundance of Equine infectious anemia virus and Escherichia virus BIFF was both increased in severe group (Fig. 4f).

### Correlation between the microbiome and clinical indexes

**Fig. 5:**
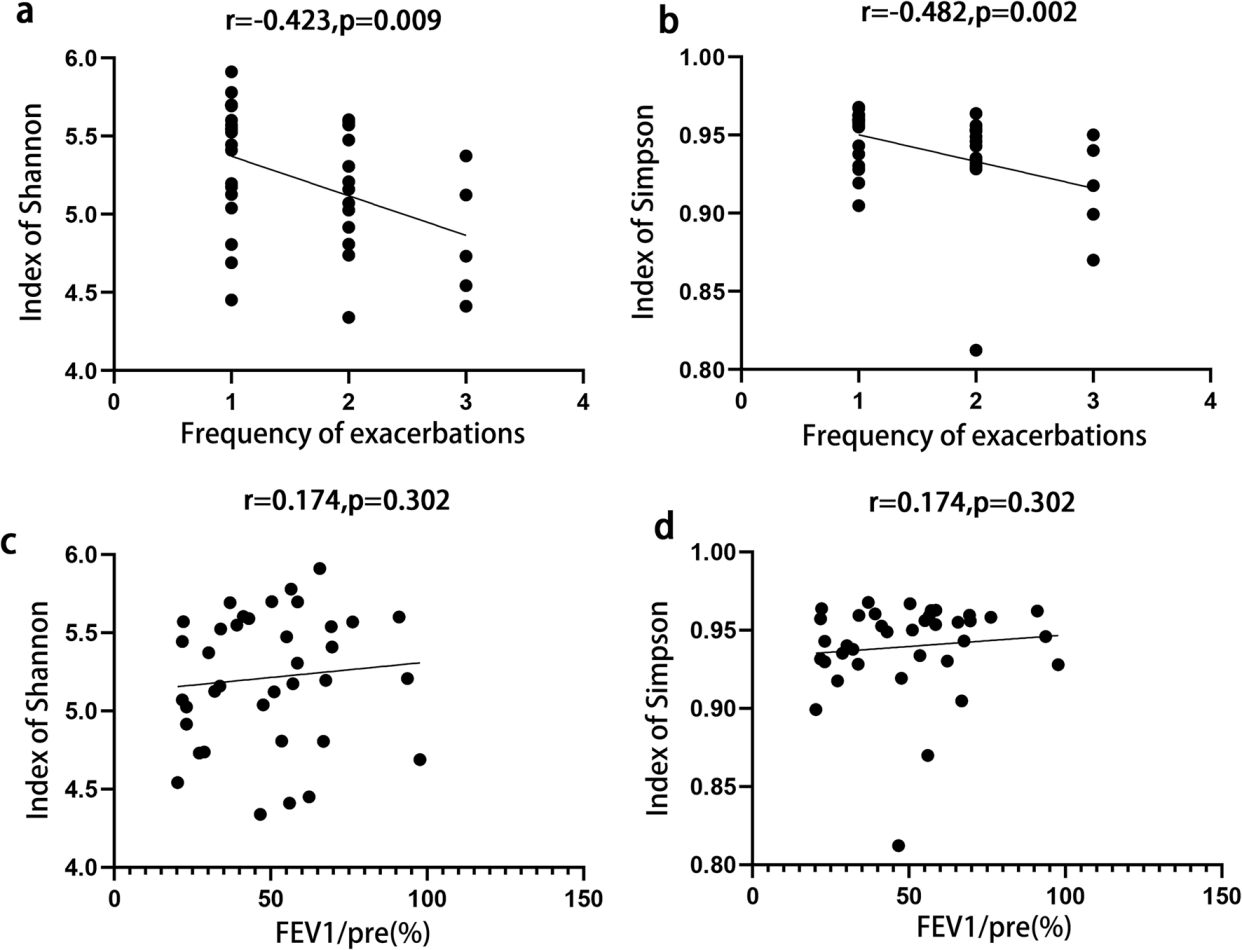
Correlation between sputum microbiome diversity and clinical variables in stable COPD. **a** Frequency of exacerbation had a negative correlation with Shannon index (r = − 0.423, p = 0.009). **b** Frequency of exacerbation had a negative correlation with Simpson index (r = − 0.482, p = 0.002). **c** FEV_1_/pre had no correlation with Shannon index. **d** FEV_1_/pre had no correlation with Simpson index

Correlation analysis was performed between clinical variables (frequency of exacerbation, age, smoking index, BMI, CAT, mMRC, FEV_1_, FEV_1_/pre (%) and FEV_1_/FVC) and microbiome diversity index (Shannon and Simpson index). Spearman’s correlation analysis showed that the frequency of exacerbation had a negative correlation with Shannon index (r = − 0.423, p = 0.009) and Simpson index (r = − 0.482, p = 0.002), and FEV_1_/pre showed no significant correlation with either Shannon or Simpson index (Fig. 5). In addition, age was also found to be negatively correlated with both Shannon and Simpson indexes (Additional file 1: Fig. S2).

**Fig. 6:**
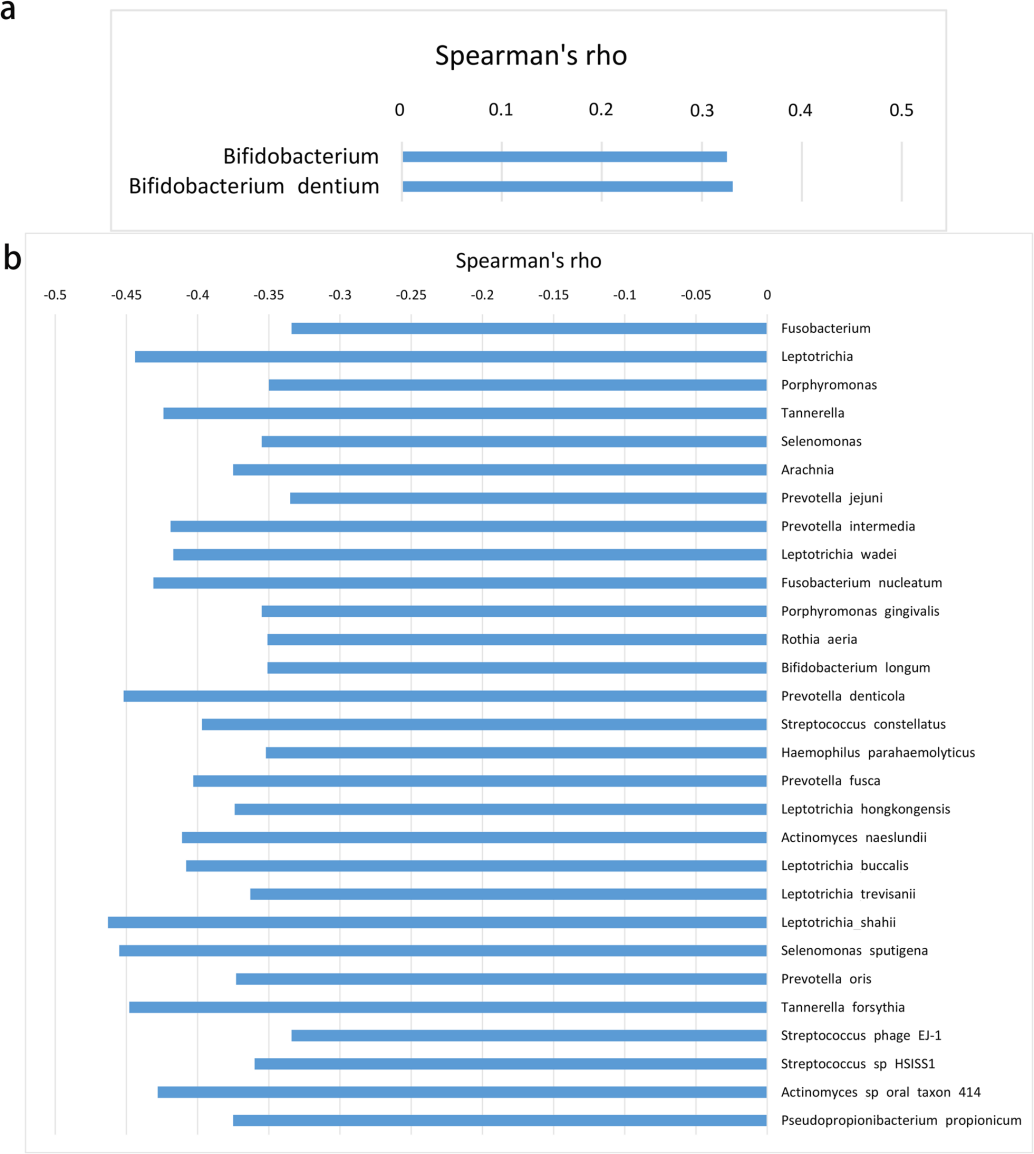
Correlation between relative abundance of the microbiome and clinical variables. **a** Bifidobacterium and Bifidobacterium dentium had a positive correlation with FEV_1_/pre (r = 0.325 or 0.331); **b** A total of 29 bacterial genera and species showed a negative correlation with the frequency of exacerbation (r ranged from − 0.463 to − 0.334)

An additional correlation analysis was performed between clinical variables (FEV_1_/pre and frequency of exacerbations) and the abundance of the microbiome genera and species that were identified by LDA analysis. Bifidobacterium and Bifidobacterium dentium proved to have a positive correlation with FEV_1_/pre (r = 0.325 or 0.331) (Fig. 6a). A total of 29 bacterial genera and species showed a negative correlation with the frequency of exacerbation(r ranged from − 0.463 to − 0.334), 79.3% of which belonged to phylum Actinobacteria, Bacteroidetes and Fusobacteria (Fig. 6b).

**Fig. 7:**
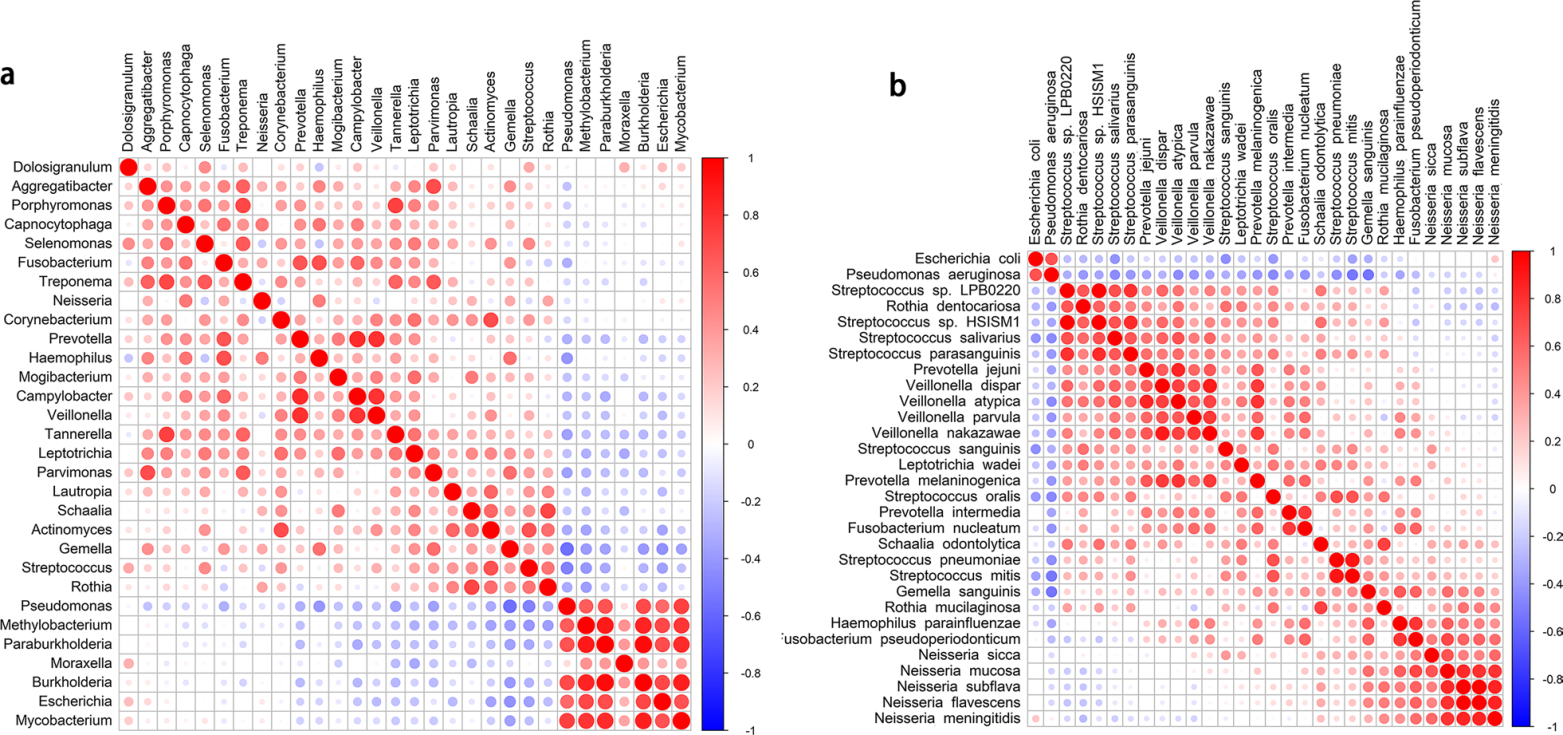
Sputum microbiome networks in stable COPD. **a** Heatmap of Spearman correlation of top 30 bacterial genera. **b** Heatmap of Spearman correlation of top 30 bacterial species

The Spearman correlations between the top 30 bacteria genus and species are shown in Fig. 7 and used to explore potential co-existence and co-exclusion relationships.

## Discussion

To the best of our knowledge, this is the first study reporting the use of mNGS to investigate the sputum microbiome in COPD of different exacerbation frequencies or lung function. The key findings of our study are as follows. First, the microbiome alpha diversity in FE group was significantly decreased as compared with NFE group, which was verified in the subsequent analysis, showing that the frequency of exacerbation was negatively correlated with microbiome diversity; microbiome diversity was similar between mild and severe groups. Second, the microbiome composition underwent significant changes. More specific bacterial taxa were enriched in NFE group and they mostly belonged to phylum Actinobacteria, Bacteroidetes and Fusobacteria. More specific bacterial taxa were enriched in mild group and they mostly belonged to phylum Actinobacteria and Bacteroidetes.

It was found in our study that microbiome alpha diversity in FE group was significantly decreased at specie level, and there was a negative correlation between the frequency of exacerbation and alpha diversity, which is consistent with the finding of Pragman et al. [13, 14], who analyzed the oral wash and sputum samples from COPD patients with 16S rRNA, and found that NFE samples were more diverse than FE samples [14]. Yang et al. [15] applied 16S rRNA to investigate the sputum microbiome of COPD outpatients, and discovered that Chao1 index and observed OTUs in high-risk exacerbator (HRE) group were significantly lower than those in low-risk exacerbator (LRE) group, while Shannon index was similar between the two group, knowing that Shannon index addresses the richness and evenness of the community, while Chao1 and Observed OTUs emphasizes the number of species. The reason for the difference may also come from different grouping criteria. In the study by Yang et al., cases of COPD with < 2 moderate exacerbation and no severe exacerbation per year were assigned to LRE group, and otherwise to HRE group. In addition, different sequencing techniques (16S rRNA vs. mNGS) may also the possible reason. It was found in our study that α diversity was similar between mild and severe groups, which is consistent with Yang’s finding [16].

Out of top 10 bacteria taxa, Veillonella, Fusobacterium and Prevotella jejuni were found to be more enriched in NFE group compared with FE group. Veillonellae are anaerobic Gram-negative cocci, usually found in the mouth, gastrointestinal tract and the vagina. Veillonella species were found to have a strong relationship with bronchoalveolar lavage (BAL) lymphocyte counts, BAL neutrophil counts and exhaled nitric oxide of early COPD patients [17]. In another study, Veillonella were found strongly associated with a Th17 lung inflammation phenotype in participants without known pulmonary disease [18, 19]. These findings seemed contrary to ours, which may due to different study population. Little was known about the association of Fusobacterium, Prevotella jejuni and COPD yet. Rothia were more abundant in mild group compared with severe group. The genus Rothia are Gram-positive, and found as colonizers of the human oral cavity. In a cohort of adults with bronchiectasis, the abundance of Rothia species was negatively correlated with pro-inflammatory markers (interleukin (IL)-8 and IL-1β) and matrix metalloproteinase (MMP)-1, MMP-8 and MMP-9 in sputum. Rothia species were also found inhibit NF-κB activation in epithelial cells [20].

Our LDA analysis revealed that many bacterial taxa were more abundant in NFE group, and they mostly belonged to phylum Actinobacteria, Bacteroidetes and Fusobacteria. Meanwhile, some bacterial taxa were more enriched in mild group, and they mostly belonged to phylum Actinobacteria and Bacteroidetes. Pragman et al. [14] also found that Actinomyces was more common in the sputum of NFE group as compared with FE group. Guo et al. [20] reported that large numbers of bacteria that colonized on the surface of the respiratory mucosa of healthy people were mainly Actinobacteria and Bacteroidete. He et al. [21] found that community-acquired pneumonia presented a depletion of some genera of the phylum Bacteroidetes in bronchoalveolar lavage samples. All these studies agree with our research in some sense. We found that species more enriched in FE or severe group mostly belonged to phylum Proteobacteria, which agrees with the finding of the over-representation of Proteobacteria phylum in severe COPD in other studies [13, 21, 22].

Bifidobacterium and Bifidobacterium dentium showed a positive correlation with FEV_1_/pre. Some studies reported that Bifidobacterium was good to airway inflammation in asthma [23, 24], which seemingly agrees with our finding. In addition, we identified a lot of bacterial genera or species mainly belong to phylum Fusobacteria, Bacteroidetes and Actinobacteria. They were found to be negatively correlated with the frequency of exacerbation, which is first reported in our study.

To the best of knowledge, this is the first study reporting the use of mNGS to compare the sputum microbiome in COPD of different exacerbation frequencies or lung function. Compared with 16S rRNA, mNGS has the advantages of (1) covering not only bacteria but also eukaryotes and DNA viruses; (2) higher taxonomic resolution at species level instead of genus level; (3) lower potential of bias due to the untargeted nature of the methodology; (4) enabling to discover new or unexpected organism [25]. As the sputum was collected during stable phase of COPD patients, the effect of antibiotics should have been avoided. Nowadays, mNGS may play a role in identifying pathogen in COPD exacerbation. Our research applied mNGS to investigate the correlation of sputum microbiome and disease severity during COPD stable phase, which means in the future mNGS may be a strong tool to predict COPD progress and direct COPD treatment.

There are some limitations in this study. Firstly, the sample size was not large enough, and therefore larger COPD cohort studies are required to verify our findings and conclusions. In addition, our study is a cross-sectional study which only allowed for correlation analysis and did not allow for causal relationship exploration. To form a longitudinal cohort, the patients enrolled in this study will be followed up continuously. Finally, the baseline FEV_1_/pre was not balanced between NFE and FE groups. Although we analyzed differences between groups with different lung functions to minimize the limitation, some bias may not be avoidable.

## Conclusion

Microbiome diversity was lower in frequent COPD exacerbators, but similar between different lung function groups. The microbiome composition changed between different groups as well. It is still necessary to gain a better understanding about the underlying mechanism of the microbiome and exacerbation frequency or lung function for the sake of improving the clinical outcome of COPD treatment.

## Supplementary Information

**Additional file 1.** Supplemental Figures.

## Data Availability

Raw sequence data have been deposited in the NCBI “Sequence Read Archive” (SRA) under the project accession PRJNA858967. Other data generated or analysed during this study are included in this published article.

## Abbreviations

COPD: Chronic obstructive pulmonary disease
mNGS: Metagenomic next-generation sequencing
FE: Frequent exacerbators
NFE: Non-frequent exacerbators
FEV_1_: Forced expiratory volume in 1 s
FEV_1_/pre: FEV_1_ percent predicted
n: Number
NGS: Next-generation sequencing
PCR: Polymerase chain reaction
16S rRNA: 16S ribosomal RNA
mMRC: Modified Medical Research Council dyspnea scale
CAT: COPD Assessment Test
NTC: Non-template controls
PERMANOVA: Permutational multi-variate analysis of variance
PCA: Principal component analysis
PCoA: Principal co-ordinates analysis
LEfSe: Linear discriminant analysis effect size
BMI: Body mass index
FVC: Forced vital capacity
HRE: High-risk exacerbator
LRE: Low-risk exacerbator
OTU: Operational taxonomic units
LDA: Linear discriminant analysis
BAL: Bronchoalveolar lavage
