# Supplementary material for: Analysis of sputum microbial metagenome in COPD based on exacerbation frequency and lung function: a case control study
Source: Respir Res. 2022 Nov 19;23:321. doi: 10.1186/s12931-022-02246-9 (PMC9675190; doi:10.1186/s12931-022-02246-9)
Supplement: Supplementary file 1 — Additional file 1. Supplemental Figures. [file 12931_2022_2246_MOESM1_ESM.docx]

Supplemental Information

Supplemental Figures


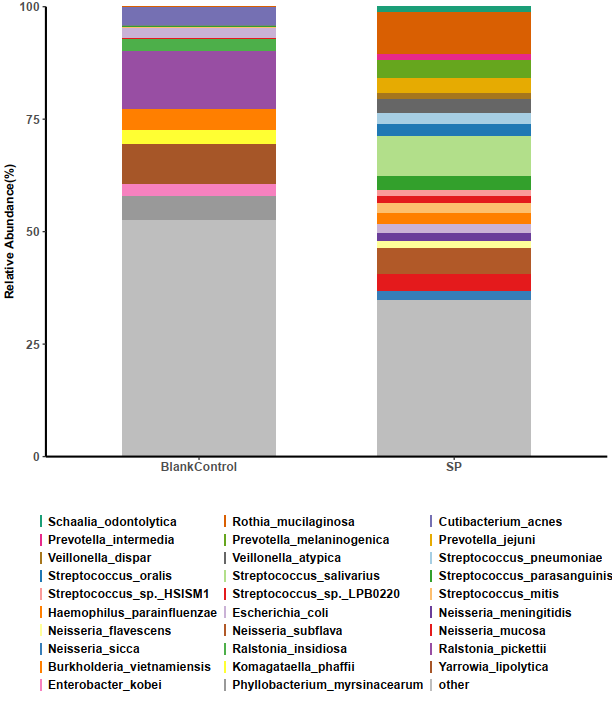


Supplemental Figure 1.

**Assessment of background DNA contamination in sequencing blank extraction controls.** Microbiome composition comparison of BlankControl group and Sample(SP) group.


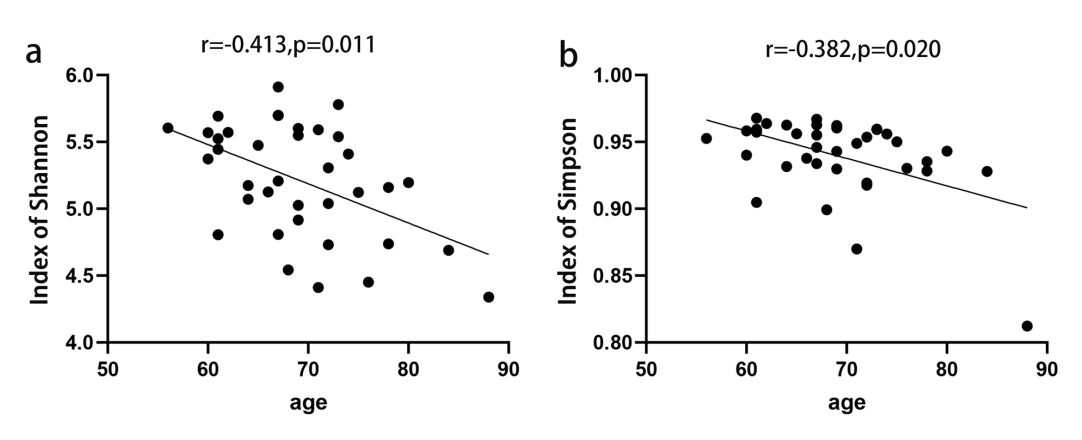


Supplemental Figure 2.

**Correlation between sputum microbiome diversity and age in stable COPD. (a)** Age had a negative correlation with Shannon index (r=-0.413, p=0.011). **(b)** Age had a negative correlation with Simpson index (r=-0.382, p=0.020).
